# An evaluation of support to the second victims in Tshwane District Health Services, South Africa

**DOI:** 10.4102/safp.v66i1.5980

**Published:** 2024-09-26

**Authors:** Doudou K. Nzaumvila, Tombo Bongongo, Indiran Govender, Sunday O. Okeke

**Affiliations:** 1 Department of Family Medicine & Primary Health Care, Faculty of Medicine, Sefako Makgatho Health Sciences University, Pretoria, South Africa

**Keywords:** patients’ safety incidents, second victims, institutional support, healthcare professionals, Tshwane District

## Abstract

**Background:**

Initiatives to reduce patient safety incidents (PSI) and support healthcare professionals who may experience psychological trauma as a result are becoming increasingly common. However, little is known about the quality of the support provided by Tshwane District Health Services. Therefore, it is necessary to assess their assistance for the second victims in order to evaluate their effectiveness.

**Methods:**

A cross-sectional study was conducted, and 319 healthcare professionals from six primary health care institutions were invited to participate in the study. The sociodemographic information, work experience, emotional support, familiarity with the concept of the ‘second victim’ and involvement with PSIs were collected.

**Results:**

The mean age was 39.8 years, ranging from 22 years to 66 years. The majority of participants were females (*n* = 249; 78.1%), nurses (*n* = 153; 49.2%), and those with 5–9 years of experience (*n* = 82; 25.8%). Most participants (*n* = 168; 52.7%) were aware of the possibilities of emotional support, while less than half (*n* = 142; 44.5%) were familiar with the term ‘second victim’. The cumulative incidence of adverse events in the institutions was 19.4%, and the majority of second victims (*n* = 39; 62.9%) emotionally felt the need to speak with someone about it, preferably outside of the workplace. Less than 5% of individuals received support that was initiated by existing structures at their workplace.

**Conclusion:**

Frameworks exist to assist second victims, although they are only known to some healthcare professionals. However, their current use in Tshwane health facilities is ineffective. After experiencing PSIs, second victims often rely on psychological assistance outside of the workplace.

**Contribution:**

Authorities need to determine the causes behind some healthcare professionals’ lack of awareness regarding the support framework for second victims, as well as their growing tendency to rely on psychologists outside of the workplace, and corrective measures should be implemented.

## Introduction

Inadequate patient safety not only places the patient at risk of an adverse event but also has a negative effect on healthcare providers (HCPs).^1^ Patient safety incident (PSI) is a sobering reality for some HCPs. It poses acute and chronic risks of psychological trauma to HCPs.^1,2^ A growing emphasis on enhancing the safety of the healthcare system has resulted in several efforts in recent years to limit PSI to unanticipated causes as much as possible. Nonetheless, there has been less focus on assisting HCPs in managing distressing patient incidents.^3,4^

Whenever a PSI occurs because of a failure of expected safety mechanisms or precautions, healthcare professionals (HCPs) may experience distress. This can be because of their involvement in, witnessing of, or failure to prevent the adverse event.^5^ This is what is considered to be the second victim.^6^ However, there is an emerging understanding that this term should not be used, particularly among patients and HCPs.^7^ Even Wu, who propounded the term in 2000, concurs.^8^ The current situation regarding second victims demonstrates ambiguity. There is sufficient evidence to indicate that psychological support to second victims dealing with the trauma of adverse events benefits the healthcare system. It not only improves the well-being of second victims but also benefits other staff members, enhancing retention and readiness to provide quality care.^8,9^ This is in contrast with the blaming and/or punitive culture which still exists in some institutions.^10,11^ Patient safety incident-related psychological and physical burdens should be addressed as they can lead to an additional PSI of future patients^10^ and bad practices, such as defensive medicine.^11,12^

Owing to decreased job satisfaction following PSI, second victims may reconsider their perceptions of professional self-efficacy,^3^ or worse, consider changing professions.^13^ A range of clinical symptoms such as fatigue, frustration, guilty feelings, insomnia and anxiety were reported.^14,15,16^ These can be classified as acute anxiety, acute and chronic post-traumatic stress disorder and suicide.^17,18^ As a result of the severity of the problem, some second victims developed substance use disorders^9^ and relied on non-work-related support^3^ as a coping mechanism. Given the statistics of 10% – 30% of patients being injured by HCP during treatment or investigations, attending to the psychological needs of second victims is viewed as a priority, as more than 50% of second victims develop emotional stress post-PSI in an already precarious profession with the risk of changing careers.^13^ Attention to second victims is sadly not optimised in South Africa in general.^3,19^ Additionally, there has never been a study on support to second victims in Tshwane health facilities to assess the level of assistance they receive. As a result, the current study evaluated the support of second victims in some of the health facilities in the Tshwane District Health Services.

## Research methods and design

This cross-sectional study was conducted in six primary health care facilities in the Tshwane District Health Services from April 2020 to July 2020. These facilities included three community healthcare centres (Clinic 1, Clinic 2 and Clinic 3), two district hospitals (Hospital A and Hospital B) and the family medicine ward at an Academic Hospital (Hospital C). The sample consisted of all HCPs, namely doctors, nurses and healthcare scientists (HCS), employed by the facilities mentioned earlier. The category HCS included audiologists, clinical associates, occupational therapists, pharmacists, physiotherapists, psychologists and radiographers. A convenient sample size of 319 participants was obtained. Participants registered with the Health Professional Council of South Africa and actively involved in patients’ care and safety, such as HCPs and HCS, are eligible to participate. Facility management personnel, however, are not eligible. The authors and additional nurses whom the authors had trained collected the data using a validated data collection form that had previously been utilised in other studies as a self-administrated anonymous questionnaire.^20^ Two researchers independently piloted the questionnaire in two primary health facilities: one community health centre and one hospital. Their feedback was used to adjust the questionnaire according to the purpose of the study and the population specificity of the study. Potential participants were voluntarily invited to participate in the study after the aim of the study had been explained to them and all participants had signed an informed consent form. The researchers and research assistants recruited the participants through word of mouth by leveraging their personal and professional networks at presentations during professional meetings.

Data were captured in a spreadsheet and then imported for analysis into Instat^R^. The results were presented in tables using descriptive analysis.

### Ethical considerations

Ethical clearance to conduct this study was obtained from the Sefako Makgatho Health Sciences University, Research Ethics Committee (SMUREC/M/197/2019: IR) and Gauteng Health Department, Tshwane Research Committee (GP_202001_048).

## Results

### Sociodemographic results

The mean age was 39.8 years with a standard deviation of 10.6 years. The youngest was 22 years old, and the oldest was 66 years. The majority of participants (*n* = 56; 33.7%) were between the ages of 30 years and 39 years. Many participants were from Hospital A (*n* = 131; 41.3%). Most of them had 5–9 years of experience (*n* = 82; 25.9%), were female (*n* = 249; 78.1%) and nearly half were nurses (*n* = 153; 49.2%). Table 1 presents more information on the sociodemographics of the participants.

**TABLE 1:** Sociodemographics of the participants.

Variable	Frequency (*n*)	Percent (%)
**Age group (years) (*n* = 166)**		
20–29	29	17.5
30–39	56	33.7
40–49	46	27.7
≥ 50	35	21.1
Total	166	100.0
**Health facilities (*n* = 317)**		
Clinics	99	31.2
Hospital C	16	5.0
Hospital B	71	22.4
Hospital A	131	41.3
Total	317	99.9
**Gender (*n* = 316)**		
Male	67	21.2
Female	249	78.8
Total	316	100.0
**Category of HCP (*n* = 319)**		
Doctor	34	10.6
Nurse	153	48.0
HCS	132	41.4
Total	319	100.0
**Years of experience (*n* = 317)**		
0–4	73	23.0
5–9	82	25.9
10–14	64	20.2
15–19	38	12.0
20–24	22	6.9
25 and more	38	12.0
Total	317	100.0

HCP, healthcare providers; HCS, healthcare scientists.

### Knowledge of institutional structured support to second victims

The findings showed that a little over half of the participants (*n* = 168; 52.7%) were aware of the possibility of emotional support at their current workplace. There was observed variability when the data were broken down by category. One would raise the number, while the other would fall below 50% because less than a quarter of the doctors (23.5%) and less than half of the nurses (47%) were aware of such support at their workplace; the HCS (66.7%) raised the figure in the category of HCPs. In terms of health facilities, Hospital A had 65.4% of HCPs, but clinics (47%), Hospital C (37.5%) and Hospital B (46.5%) all had less than half of HCP awareness of the available assistance.

When years of experience were considered, there was a gradual and consistent increase of awareness among HCPs with years of experience to reach a summit of 68.8% in the group of 15–19 years of experience, followed by an irregular decrease. In addition, HCPs with less than 5 years of experience and those with more than 25 years of experience were aware of the possibility of support in 37% and 31.6% of cases, respectively, indicating declines in these extreme years of experience.

Table 2 contains more facts on awareness of institutional structured support to second victims.

**TABLE 2:** Knowledge of existing support, familiarity with second victims and prevalence of patient safety incidents.

Variables	At my workplace, there is a well-structured organisation to support HCPs involved in PSI		I am familiar with the term ‘second victim’		I have been involved in PSI at my current workplace	
	*n*	%	*n*	%	*n*	%
**Category of HCP (*n* = 319)**						
Doctor (*n* = 34)	8	23.5	11	32.4	11	32.4
Nurse (*n* = 153)	72	47.0	48	31.4	31	20.3
HCS (*n* = 132)	88	66.7	52	34.0	20	15.2
Total	168	52.7	111	34.8	62	19.4
**Years of experience (*n* = 317)**						
0–4 (*n* = 73)	27	37.0	25	34.2	16	21.9
4–9 (*n* = 82)	48	57.1	32	38.1	16	19.5
10–14 (*n* = 64)	42	66.7	18	28.6	9	14.0
15–19 (*n* = 38)	26	68.4	20	52.6	6	15.8
20–24 (*n* = 22)	13	59.1	9	40.9	6	27.3
≥ 25 (*n* = 38)	12	31.6	2	5.3	9	23.7
Total	168	52.8	106	33.4	62	19.5
**Health facilities (*n* = 317)**						
Clinics (*n* = 99)	41	41.0	20	20.2	13	13.1
Hospital C (*n* = 16)	6	37.5	5	31.3	3	18.8
Hospital B (*n* = 71)	33	46.5	26	36.6	20	28.2
Hospital A (*n* = 131)	87	65.4	61	45.6	26	19.8
Total	167	52.2	112	35.3	62	19.4

HCP, healthcare providers; PSI, patient safety incidents.

### Healthcare providers’ familiarity with the term second victim

Analysis indicated that almost one-third of participants in different categories were familiar with the term ‘second victim’, which refers to HCPs who have experienced psychological PSIs. When groups in different categories were analysed, it was found that 52.6% of HCPs with 15–19 years of experience were familiar with the terminology relating to second victims. Table 2 provides additional information.

### Prevalence of patient safety incidents

Hospital B had the highest PSI prevalence at 28.2%, compared to the overall prevalence of 19.4% across all facilities. The most common HCPs for PSI were doctors (32.4%), followed by nurses (20.3%) and those with 20–24 years of experience (27.3%). Table 2 provides additional details.

### Need for emotional support among second victims

Our research revealed that most HCPs who were second victims of PSI felt the need to talk about the incident (62.9%). Doctors (72.7%) and HCPs with more than 25 years of experience (75%), respectively, accounted for the highest percentages. Table 3 offers additional information.

**TABLE 3:** Emotional support needs among second victims.

As a second victim, I did feel the emotional need to talk about the event	Yes		No	
	*n*	%	*n*	%
**Category of HCP (*n* = 62)**				
Doctor (*n* = 11)	8	72.7	3	27.3
Nurse (*n* = 31)	19	61.3	12	38.7
HCS (*n* = 20)	12	60.0	8	40.0
Total	39	62.9	23	37.1
**Years of experience (*n* = 61)**				
0–4 (*n* = 16)	10	62.5	6	37.5
4–9 (*n* = 16)	10	62.5	6	37.5
10–14 (*n* = 9)	6	66.7	3	33.3
15–19 (*n* = 6)	3	50.0	3	50.0
20–24 (*n* = 6)	4	66.7	2	33.3
≥ 25 (*n* = 8)	6	75.0	2	25.0
Total	38	62.3	23	37.8
**Health facilities (*n* = 60)**				
Clinics (13)	9	69.2	4	30.8
Hospital C (3)	3	100.0	0	-
Hospital B (20)	11	55.0	9	45.0
Hospital A (24)	16	66.7	8	33.3
Total	39	65.0	21	35.0

HCP, healthcare providers; HCS, healthcare scientists.

### Emotional support to second victims

Although most second victims needed to talk about their feelings, it was observed that many discussed it with a friend at their workplace (35.9%), but mainly did so outside of the workplace (64.1%). Some participants (10.3%) mentioned debriefing and validation of feelings as the only form of support among many other possible forms. This was mentioned only by nurses (10.5%) and HCS (16.7%). Table 4 provides more details.

**TABLE 4:** Emotional support to second victims.

I discussed my emotional feelings about the adverse event with	Doctor		Nurse		HCS		Total	
	*n*	%	*n*	%	*n*	%	*n*	%
Colleague in a different profession (*n* = 0)	0	-	0	-	0	-	0	-
Colleague in the same profession (*n* = 0)	0	-	0	-	0	-	0	-
Friend in the unit at my workplace (*n* = 14)	2	25.0	8	42.1	4	33.3	14	35.9
Organisational structure at my workplace (*n* = 0)	0	-	0	-	0	-	0	-
Supervisor (*n* = 0)	0	-	0	-	0	-	0	-
I discussed my emotional feelings outside my workplace	6	75.0	11	57.9	8	66.7	25	64.1
**After the adverse event, did someone in your facility offer you the following?**								
Debriefed or validated feelings	0	-	2	10.5	2	16.7	4	10.3
Listened actively to you	0	-	0	-	0	-	0	-
Proposed solutions to the current event or ways to prevent similar events	0	-	0	-	0	-	0	-
Provided a safe and friendly space to talk	0	-	0	-	0	-	0	-
Provided an opportunity to process the event	0	-	0	-	0	-	0	-
Reassurance	0	-	0	-	0	-	0	-
Reinforced the idea that events are part of the profession or learning	0	-	0	-	0	-	0	-
Shared similar experiences	0	-	0	-	0	-	0	-
Showed compassion	0	-	0	-	0	-	0	-
Other	0	-	0	-	0	-	0	-

HCS, healthcare scientists.

## Discussion

This study on the second victims of PSI highlighted some aspects of the phenomenon in the Tshwane District, which will be articulated in the discussion. The PSI literature has very little information on the prevalence of second victims, but the little information available shows that it ranges widely from 14.3% to 59%^1,17,21^ and can even fluctuate, depending on time and place.^22^ We found that 19.4% of HCPs, primarily more doctors than nurses, had PSI at their current place of employment at least once. The incidence of PSI is usually patient-centred. Mgobozi^19^ and colleagues found 4.12 PSIs per 10 000 inpatient days. However, Gqaleni^23^ and colleagues in their study classified PSI incidence into different categories. This dearth of second victim incidence reports would suggest that the current state of affairs is inadequate for handling second victims. The main focus of the literature is on how they deal with situations rather than the statistical incidence among HCPs. This is a missed opportunity because it could have been used to identify survivors who could debrief or validate feelings, provide a safe and friendly space to talk, give others a chance to process the event, reassure them that such things happen in the course of learning and practice, share similar experiences or demonstrate compassion towards others within the same institution. Considering that nearly half of HCPs would become a second victim at least once during their career,^24^ the lack of incidence reports on second victims is astounding.

Given the fact that PSI is a daily occurrence for HCPs^25^ and considering that it can happen in any HCP carrier,^20,25^ one would anticipate that HCPs would be aware of where to resort to for help. Our results showed that 52.7% of participants were aware of the opportunity for emotional support from a well-structured organisation to assist HCPs involved in PSI at their current workplace. As PSI is evaluated by a quality assurance office in every healthcare facility, a higher percentage than that indicated earlier should have been projected. This may be understood from the findings by Mayeng,^26^ which revealed that certain HCPs had unfavourable perceptions of all the safety dimensions. This can be a result of the inadequate way some HCPs understand PSI.^26^ To add to the negative perceptions of patient safety culture in general by HCPs, the blaming perception around second victims, in particular, could be a part of the problem. A comprehensive quality reform initiative that considers patients, their families and HCPs is required to reduce PSI and its consequences on HCPs, and significant transformational leadership is also required to create a culture free of blame and a learning environment where PSI can be improved.^19^

This change ought to be thorough enough to incorporate the contentious term ‘second victim’, which is used to describe HCPs involved in PSI. Our findings revealed that 34.2% of participants were not acquainted with this term, even though it is currently becoming widely used in both scientific and political fields.^7,9^ Stramez^17^ reported, however, that Bushc^17^ and Ganal found different figures at 90%. The fact that HCPs were not familiar with the term did not have any influence on their being aware of the possibility of psychological issues after PSI. This should feed the debate on the relevance of the term. Clarkson^9^ argued that the necessity to support HCPs who have been involved in PSI has never been denied, but the term ‘second victim’ was said to look like it subtly spread the idea that patient harm is random, the result of bad luck and, essentially, unpreventable. Referring to themselves as victims obscures the fact that HCPs and systems can become agents of harm, but it poses a threat to enacting the profound cultural changes required to achieve a patient-centred environment focussed on patient safety.

Our research found that 62.9% of second victims felt an emotional desire to discuss the incident, but that they did not do so at work. This should be understood in the individual’s coping mechanism and the availability of professionalism in emotional support.

### Strengths and limitations

This is the first study to analyse the prevalence and the quality of support provided to second victims in the Tshwane District, setting the path for future research in this area. Furthermore, the self-administered questionnaire permitted participants to respond freely, but the potential to generalise the findings was impeded because not all healthcare professionals in the two Tshwane regions participated.

## Conclusion

In the various healthcare facilities of the Tshwane District Health Services of South Africa, where the study was carried out, participants agreed to the need to vent their emotions after PSI. However, less support was offered at the workplace, and as such, HCPs relied on friends and other structures outside of the workplace, where they typically found a source of more support than the facility management and colleagues.

### Recommendation

Patient safety incident is a daily challenge at any health facility; various efforts are made to reduce it as much as possible. Given that it is predicted that every HCP may become a second victim at least once throughout their career, sufficient mechanisms such as awareness of the structured support and initiated psychological support from the management should be put in place to support HCPs in Tshwane.
